# New Monoclonal Antibodies for a Selective Detection of Membrane-Associated and Soluble Forms of Carbonic Anhydrase IX in Human Cell Lines and Biological Samples

**DOI:** 10.3390/biom9080304

**Published:** 2019-07-25

**Authors:** Dovile Stravinskiene, Aiste Imbrasaite, Vilma Petrikaite, Daumantas Matulis, Jurgita Matuliene, Aurelija Zvirbliene

**Affiliations:** 1 Institute of Biotechnology, Life Sciences Center, Vilnius University, Sauletekio al. 7, LT-10257 Vilnius, Lithuania; 2 Faculty of Pharmacy, Lithuanian University of Health Sciences, A. Mickeviciaus g. 9, LT-44307, Kaunas, Lithuania

**Keywords:** monoclonal antibodies, human carbonic anhydrase IX, immunoassays, cancer biomarkers

## Abstract

Monoclonal antibodies (MAbs) selectively targeting tumor-associated antigens such as carbonic anhydrase IX (CA IX) can significantly contribute to research, diagnostics, and treatment of CA IX-related cancers. CA IX is overexpressed in numerous hypoxic cancers where it promotes tumor progression. Therefore, it is considered as a promising tumor biomarker. A novel collection of MAbs against recombinant CA IX was developed and evaluated in different immunoassays for studying CA IX expression. The reactivity of MAbs with native cell surface protein was confirmed by flow cytometry and the presence of hypoxia-inducible CA IX was investigated in several human cancer cell lines. In addition, the applicability of MAbs for visualization of CA IX-positive tumor cells by immunofluorescence microscopy was demonstrated. MAb H7 was identified as the most promising MAb for different immunoassays. It recognized a linear epitope covering CA IX sequence of 12 amino acid residues 55-GEDDPLGEEDLP-66 within the proteoglycan domain. The MAb H7 was the only one of the collection to immunoprecipitate CA IX protein from cell lysates and detect the denatured CA IX with near-infrared fluorescence Western blot. It was also employed in sandwich enzyme-linked immunosorbent assay to detect a soluble form of CA IX in growth medium of tumor cells and blood plasma samples. The diagnostic potential of the MAb H7 was confirmed on formalin-fixed and paraffin-embedded tissue specimen of cervical carcinoma in situ by immunohistochemistry. The generated MAbs, in particularly clone H7, have great potential in diagnostics and research of CA IX-related cancers.

## 1. Introduction

Cancer encompasses serious diseases which are characterized by changes in the cell condition emerged from the alterations in expression of various genes and their coding proteins. Relatively unlimited and uncontrolled proliferation along with improved survival potential of cancer cells disturbs normal homeostasis of various tissues and organs leading to their failure, which deteriorates the life quality of cancer patients and, in many cases, leads to death [1,2,3]. Cancer-specific proteins that are affected by or contribute to malignant transformation are monitored to provide insights for early cancer detection, differentiation of cancer stages, patient prognosis determination, prediction of response to therapy, and treatment selection [4,5,6]. The growing demand for the advanced technologies and novel biological tools for screening and treatment of cancer patients caused intensive development in fields of immunodiagnostic and immunotherapy [7,8]. Monoclonal antibodies (MAbs) developed to recognize tumor-specific antigens are considered as core agents in many diagnostic methods such as enzyme-linked immunosorbent assay (ELISA), immunohistochemistry (IHC), or positron-emission tomography to analyze serum, saliva, sputum, urine and tissues both ex vivo and in vivo [9,10,11]. Antibody-based immunotherapy use antibodies or their derivatives as mediators between the cancer antigen and the toxic substance, such as chemotherapy drugs or radioactive particles, or components of the host′s immune system capable of destroying the cancerous cell [12]. The major difficulty of antibody-based cancer diagnostics or therapy relates to the identification and validation of reliable tumor antigens. Studies are focusing on proteins expressed entirely by tumor cells with no or minimal expression in normal tissues to overcome ambiguous or insignificant results when considering identification of pathology as well as emergence of off-target toxicities in case of immunotherapy [7,8].

Two dimeric transmembrane isozymes of carbonic anhydrase (CA) family carbonic anhydrase IX (CA IX) and carbonic anhydrase XII (CA XII) are involved in carcinogenesis by contributing to extracellular pH regulation under hypoxia conditions thus promoting tumor cell growth and survival. Active centers of both cancer-associated CAs are directed to the outside of the cell which is the most important feature to apply antibody-based detection or therapy [13,14,15]. The expression pattern of CA XII is less appropriate in the context of immunotherapy, as this enzyme is found in various normal tissues [16]. In contrast, CA IX is present only in normal small intestine, pancreas, and gastric and biliary mucosa, while it is overexpressed in many tumors like renal, bladder, colon, breast, cervix, ovary, head and neck, or lung [17,18,19]. The significance of CA IX in malignant processes, its diverse expression in normal and cancerous tissues and external localization in cells makes CA IX an attractive tumor antigen, which diagnostic and therapeutic relevance is under investigation [4,14,20].

First MAbs against CA IX were generated using tumor cells as an immunogen without identifying the target. Mice were immunized with cell homogenates of either primary renal cell carcinoma lesions, which resulted in the MAb G250 [21], or cervical adenocarcinoma cell line (HeLa) cells which resulted in the MAb M75 [22]. It was not until 1996 that the antigen recognized by the MAb M75 was demonstrated to be CA IX [23,24]. Four years later, CA IX was also identified as a target of the MAb G250 [25]. Despite the lack of knowledge about molecular characteristics of G250 antigen, the first clinical trials based on MAb G250 were performed already in 1993 for human renal cell carcinoma treatment, which were soon replaced by clinical studies using chimeric G250 antibody [26]. The MAb G250 does not inhibit CA IX, however its chimeric derivate with immunoglobulin G (IgG) constant domain replaced to human IgG1 sequence can trigger antibody-dependent cellular cytotoxicity thus mediating tumor cell killing [27,28]. Well characterized MAb M75 targeting the proteoglycan (PG) region of CA IX was involved in many studies, mainly as a primary antibody in the IHC for the detection of CA IX in normal or tumor tissues [29,30]. It was also employed as a detection antibody in sandwich ELISA to analyze body fluids of cancer patients for the presence of a shedded extracellular domain of CA IX [31,32]. Later, more antibodies against CA IX were generated, in particular for therapeutic purposes or in vivo visualization. Recombinant human antibodies and antibody fragments (single-chain fragment variable, small immunoprotein formats, and antigen-binding antibody fragment) derived by phage-display technology were reactive with native CA IX protein on cell surface, had an inhibitory effect, or induced surface CA IX internalization efficiently. Future prospects to use these antibodies for diagnostics and therapy of CA IX-positive cancers are intensively investigated [33,34,35].

Despite previously produced antibodies, there is still a high demand of new antibodies against CA IX to overcome some existing limitations. Only a few of antibodies mentioned above are comprehensively characterized with defined epitope localization or a broad analysis for their applicability in various immunological methods. Here we describe a novel collection of extensively characterized MAbs against human CA IX generated after immunization of mice with either recombinant full-length extracellular domain of CA IX or recombinant catalytic domain without the PG region. IgG-secreting hybridoma cells were selected with a fully automated cell cloning system which resulted in highly specific MAbs against CA IX. In particular, the MAb of clone H7 has many application possibilities for the detection of both denatured and native CA IX protein in cell lines and human tissues, as well as soluble form of CA IX shedded into the growth medium of cells or human blood, as demonstrated by different immunological methods.

## 2. Materials and Methods

### 2.1. Cell Lines

Mouse Sp2/0 myeloma cells (ATCC, CRL-1581) were used as a fusion partner for production of hybridomas. Cells were cultured in complete Dulbecco′s modified Eagle′s growth medium (DMEM, Merck KGaA, F 0445, Germany) supplemented with 2 mM L-glutamine (Merck KGaA, G7513), 200 μg/mL gentamicin (Merck KGaA, A 2712) and 9% fetal bovine serum (FBS, Merck KGaA, S 0615) in humidified atmosphere at 37 °C and 5% CO_2_. Low passage Sp2/0 cells were maintained by replacement of medium every 2 to 4 days depending on cell density to have sufficient quantity of high viability cells in the log phase at the time of collection for fusion.

Human tumor cell lines used to investigate MAb specificity were obtained from ATCC: A549 (CCL-185), A498 (HTB-44), Jurkat (TIB-152), HeLa (CCL-2), A431 (CRL1555), CaSki (CRL-1550), MCF-7 (HTB-22), and U-87 (HTB-14). Cells were cultured in RPMI-1640 growth medium (Merck KGaA, FG 1215) supplemented with 2 mM L-glutamine, 200 μg/mL gentamicin, and 10% FBS. Cells were grown in normoxic (at 37 °C, 5% CO_2_, and 21% O_2_) and hypoxic (at 37 °C, 1% O_2_, 5% CO_2_, 94% N_2_) conditions in NUAIRE 5731 incubator with hypoxic (O_2_) control.

Human triple-negative breast cancer cells MDA-MB-231 for immunofluorescence staining or near-infrared fluorescence were obtained from Prof. Rytis Prekeris, Colorado University. The cells were cultured in DMEM (Corning, 10-017-CV, USA) supplemented with 10% FBS (GE Healthcare Life Sciences HyClone Laboratories, SH30071, USA), 1% penicillin/streptomycin (GE Healthcare Life Sciences HyClone Laboratories, SV30010), human recombinant insulin at 1 µg/mL (Gibco, 12585014, USA), 1% MEM Sodium pyruvate (Gibco, 11360-070), and 1% MEM nonessential amino acids (Gibco, 11140050). The cells were maintained at 37 °C in a humidified atmosphere containing 5% CO_2_. To achieve hypoxia conditions, cells were grown for 2 days in a hypoxia chamber at 37 °C in a humidified atmosphere containing 5% CO_2_ and 1% O_2_. All cell cultures routinely were grown to 70% confluence and trypsinized with 0.05% Trypsin-EDTA solution (Gibco, 25300054) before passage.

### 2.2. Recombinant Carbonic Anhydrases

Recombinant proteins, corresponding to the catalytic domain of CA IX with or without the PG domain (CA IX^+PG^ and CA IX^−PG^, respectively), were used as antigens for immunization. CA IX^+PG^ (aa 38 – 414) and CA IX^−PG^ (aa 138 – 390) proteins were produced in HEK 293 cells using FreeStyle MAX 293 Expression System from Invitrogen Thermo Fisher Scientific (K900010, USA) as described previously [36,37].

Recombinant antigens for MAb cross-reactivity analysis (CA I, II, IV, VB, VI, VII, XIII, and XIV) were produced using *E. coli* expression system as described previously [36], while CA XII was produced in mammalian cell culture [38].

### 2.3. Blood and Tissue Samples

Fourteen blood samples were collected in National Cancer Institute of Lithuania. Blood samples were collected in vacutainers containing EDTA (367525, BD Biosciences, USA) and plasma samples were prepared within 24 h after collection by centrifuging for 15 min at 1000× *g*. The supernatant was transferred to another sterile tube and centrifuged again at 2000× *g* for 10 min. Plasma aliquots were stored at ≤ −70 °C.

The formalin-fixed and paraffin-embedded tissue specimens of cervical carcinoma in situ were collected at the National Cancer Institute of Lithuania after conization which was a part of the general treatment of cervical carcinoma patients. All individuals included in the study provided written informed consent prior to their inclusion and the study was approved by the Bioethics Committee of Lithuania (Permission #L-16-02/1).

### 2.4. Generation of Monoclonal Antibodies against Human CA IX Protein

Mice were maintained at the breeding colony of the Centre for Innovative Medicine (Vilnius, Lithuania) under standard animal housing conditions. Animal housing, care, and experimental protocols were performed by certified staff in accordance with FELASA guidelines and conformed to Lithuanian and European legislation (License No. LT-59-902, Permission No. 184 for breeding of mice, and Permission No. 209 for generation of polyclonal and monoclonal antibodies).

Hybridoma cell lines were generated according to the protocol based upon the procedures described by Kohler and Milstein [39,40]. Two groups of three BALB/c female mice (6–8 weeks old) received first intravenous injections of 50 µg of CA IX^+PG^ or CA IX^−PG^ antigen emulsified in an equal volume of complete Freund′s adjuvant (Merck KGaA, F5881). The following immunizations were administered with 50 µg of same antigen mixed with equal volume of incomplete Freund′s adjuvant (Thermo Fisher Scientific, 77145, USA) after 4 weeks (second immunization) and diluted in phosphate-buffered saline (PBS) after 8 weeks (third immunization). Mouse blood samples were collected before each immunization by tail bleeding and antisera tested for the presence of antigen specific antibodies by an indirect ELISA. The best responder mouse of each group was boosted with 50 µg of CA IX^+PG^ or CA IX^−PG^ protein dissolved in PBS.

Three days after the last injection, spleen cells were fused with high viability Sp2/0 cells in the presence of polyethylene glycol 4000 (Merck KGaA, P7181) at a ratio 5:1. Cells were diluted to the final density of 0.29–0.33 × 10^6^ cells/mL and cultured in DMEM medium, containing hypoxanthine/aminopterin/thymidine (HAT, Merck KGaA, H0262), 2 mM L-glutamine, 200 μg/mL gentamicin, and 15% FBS for three days in 75 cm^2^ cell culture flasks for the selection of hybrid cells. Three days later hybridomas were collected by centrifugation and transferred to semisolid growth medium for Hybridomas-Mielomas (Molecular Devices, K8867, USA) supplemented with HAT, gentamicin and CloneDetect fluorescein-conjugated agent (Molecular Devices, K8220) for the detection of IgG class MAbs and seeded to 6-well plates (2 mL/well) at an average concentration of 150,000 cells/mL. Plates were incubated at 37 °C, 5% CO_2_ with high humidity for 10 days without moving to prevent cell scattering. Before procedure for hybridoma clones isolation, the ClonePix 2 system (Molecular Devices) was sanitized and calibrate according to manufacturer’s recommendations. Clones in the semisolid media were imaged under white light and fluorescence. The individual clones with high exterior fluorescent intensity were picked by ClonePix 2 system and transferred automatically to the 96-well plate (TPP, 92096, Switzerland), containing DMEM growth medium on macrophage feeder layer. After three days, hybridomas were tested for specificity to their antigens by an indirect ELISA. Clones secreting MAbs specific to CA IX^+PG^ or CA IX^−PG^ were subcloned by limiting dilution to ensure monoclonal stability. Stable hybridomas were expanded in DMEM growth medium to produce secreted MAbs and were frozen in liquid nitrogen for long storage.

### 2.5. Enzyme-Linked Immunosorbent Assay

Indirect, direct, competitive, and sandwich ELISA formats were used to characterize the generated MAbs against human CA IX.

#### 2.5.1. Indirect ELISA

An indirect ELISA was used for the analysis of blood samples of the immunized mice, selection of anti-CA IX secreting hybridomas, and characterization of MAb specificity and affinity. Recombinant CA IX^+PG^ and CA IX^−PG^ proteins were used as antigens for coating of 96-well polystyrene plates MaxiSorp (Nunc Thermo Fisher Scientific, 44-2404-21) diluted in PBS to a concentration of 5 µg/mL (50 µL per well) and incubated overnight at 4 °C. Next, antigen immobilization plates were blocked with 200 µL/well of 2% bovine serum albumin (BSA, Merck KGaA, 05482) solution in PBS for 1 h at room temperature (RT) following a series of washes with distilled water. Samples of mice blood (1:200–1:300 dilution), hybridoma growth medium (nondiluted or 1:30 dilution), or purified antibodies (1–10 μg/mL) were diluted in PBS-T buffer (0.1% Tween 20 (Carl Roth, 9127.1, Germany) in PBS), added to the wells (50–150 µL/well), and incubated for 1 h at RT. Afterwards plates were rinsed 5 times with PBS-T buffer and drained by gentle tapping. Plates were then incubated with 50 µL/well of secondary goat anti-mouse IgG antibody conjugated to horseradish peroxidase (HRP, Bio-Rad, 1721011, USA) diluted 1:5000 in PBS-T buffer for 1 h at RT following the wash step. For signal detection, 50 µL/well of 3,3′,5,5′-tetramethylbenzidine (TMB) substrate (Clinical Science Products, 01016-1, USA) was added to the plates and incubated for 5 to 15 min at RT in the dark. The reaction was stopped by adding 25 µL/well of 3.6% sulfuric acid. The OD was measured at 450 nm (reference filter 620 nm) by a plate spectrophotometer (Multiscan GO, Thermo Fisher Scientific, 51119200).

#### 2.5.2. Direct ELISA

A direct ELISA was used for testing the activity of MAb-HRP conjugates. Antigen coating, blocking, wash steps and signal detection was performed same as described above. Conjugates were diluted with PBS-T buffer in a series from 1:100 to 1:12,800 by the dilution factor 2, adding 100 μL of each dilution per well and incubated for 1 h at RT. After washing, plates were incubated with TMB for 10 min following reaction stopping and absorbance reading.

#### 2.5.3. Competitive ELISA

Competitive ELISA was used to determine if the MAbs compete for a binding site on CA IX. Antigen coating, blocking, wash, and signal detection steps were done as in an indirect ELISA. The immobilized antigen was first incubated with the unlabeled MAbs using hybridoma growth medium diluted 1:30 in PBS-T buffer (50 µL/well) incubated for 1 h at RT. HRP-MAb conjugates diluted in PBS-T buffer were then added to the hybridoma growth medium and further incubated for 1 h at RT with gentle shaking. Appropriate dilutions (1:100–1:1000) of HRP-MAb conjugates were determined by a direct ELISA. For signal detection, TMB substrate was added and incubated for 10 min.

#### 2.5.4. Sandwich ELISA

Sandwich (two epitope) ELISA was used to detect soluble CA IX by using two MAbs directed to different epitopes of CA IX. The pair of the capture and the detection MAbs was determined using recombinant CA XI^+PG^ protein as standard diluted in PBS-T buffer to the final concentration ranging from 3000 ng/mL to 0.0169 ng/mL. Capture antibodies diluted in PBS to a final concentration of 5 µg/mL were immobilized in 96-well polystyrene plates MaxiSorp (50 µL/well) and incubated overnight at 4 °C. Blocking, wash and signal detection steps were similar to the indirect ELISA. Plates coated with capture antibodies were then incubated with diluted standard (50 µL/well) for 1 h at RT. After incubation and wash steps, the HRP labeled MAbs diluted 1:100–1:1000 in PBS-T buffer were added and incubated for 1 h at RT. The signal was developed by adding TMB substrate and incubating for 10 min. After selecting the best pair of MAbs and optimal conditions of sandwich ELISA, the assay was tested for detection of soluble CA IX in cell culture supernatants and human blood plasma samples. The sensitivity or minimum detectable dose was calculated from thirty assays as described previously [41].

### 2.6. Isotype Determination of the MAbs

The Mouse Immunoglobulin Isotyping ELISA Kit (BD Biosciences, 550487, San Diego, CA, USA) was used to determine the subtypes of the MAbs according to the manufacturer′s protocol.

### 2.7. Epitope Mapping with Overlapping Biotinylated Peptides

Biotin-labeled overlapping peptides (*n* = 15) spanning PG domain of CA IX (38–112 amino acids according to UniProtKB - Q16790) were synthesized by Pepscan (Netherlands). A linker, –SGSG–, was inserted between biotin and the 15 aa peptide (Table 1). In addition, nine 10-mer peptides to cover 52–72 amino acids sequence of PG domain and shortened peptides of possible epitope were also synthetized. Indirect ELISA was applied for epitope mapping by immobilizing 50 μL/well of avidin (Thermo Fisher Scientific, 1405-69-2) diluted with distilled water at 5 μg/mL final concentration on the bottom of MaxiSorp plates and incubating over the night at 37 °C uncovered to evaporate the liquid. The next day plates were blocked with 200 µL/well of 2% BSA in PBS. After incubation and washing, 50 μL/well of biotinylated peptides, diluted with PBS-T at 10 μg/mL final concentration, were added to the plates and incubated for 1 h at RT. Growth medium of hybridoma cells was diluted 1:50 in PBS-T and incubated for 1 h at RT. Following steps were performed as described above.

### 2.8. Purification of the MAbs from Hybridoma Supernatants

MAbs were purified from hybridoma supernatants using affinity chromatography on protein A Sepharose. A chromatography column was packed with rProtein A Sepharose Fast Flow (GE Healthcare Life Sciences, 17127901), equilibrated with washing buffer (1.5 M glycine (Carl Roth, 3908.3), 3 M NaCl (Merck KGaA, 7647-14-5), pH 8.9), and loaded with hybridoma supernatant diluted with washing buffer at 1:3 ratio. Ten column volumes of washing buffer were used to wash the column, followed by elution of bound antibodies with elution buffer (100 mM glycine, pH 3.0). Eluted antibodies were dialyzed against PBS buffer overnight at 4 °C. The purified MAbs were sterile-filtered and stored at 4 °C.

### 2.9. Conjugation of the MAbs to Horseradish Peroxidase

Purified MAbs were labeled with HRP. MAbs were dialyzed against sodium carbonate (Carl Roth, P028.1) buffer (pH 9.5) overnight at 4 °C. Peroxidase (Merck KGaA, P8375) and 0.2 M sodium periodate (Merck KGaA, 71859) were mixed together and incubated for 30 min at RT in the dark. The activated peroxidase was desalted using Sephadex G-25 column (GE Healthcare Life Sciences, 17085101), mixed with each dialyzed MAbs and incubated for 2 h at RT in the dark. The pH of 9.5 was attained by adding 0.2 M sodium carbonate buffer to the mixture. Sodium borohydride (Merck KGaA, 213462) was added to the final concentration 0.2–0.4 mg/mL and incubated for 2 h at 4 °C in the dark. The mixture was then dialyzed against PBS overnight at 4 °C. After dialysis, HRP-labeled MAbs were supplemented with BSA and glycerin (Carl Roth, 3783.1) to the final concentrations of 2% and 50%, respectively, and stored at −20 °C.

### 2.10. Determination of the Apparent Dissociation Constant

K_d_ of the MAbs was determined by an indirect ELISA. The plates were coated with CA IX^+PG^ and CA IX^−PG^ antigens and incubated for 1 h at RT with the purified MAbs at different concentrations ranging from 3.3 × 10^−8^ M to 1.863 × 10^−13^ M. The K_d_ value was calculated from titration curve using data analysis and graphing software (OriginPro 9.1, OriginLab).

### 2.11. Sodium Dodecyl Sulfate–Polyacrylamide Gel Electrophoresis

Protein samples (either 1 µg of recombinant proteins or 30 µg of cell lysates per lane) were separated by electrophoresis using 12% concentrating SDS polyacrylamide gels. The samples were prepared by adding Pierce Lane Marker Reducing Sample Buffer (Thermo Fisher Scientific, 39000, Rockford, IL, USA) and boiling for 10 min. Prestained Protein Ladder (10 to 180 kDa, Thermo Fisher Scientific, 26616) was used as size standard. After the electrophoresis (Minigel-Twin 846-010-100 and Multigel 846-010-200, Biometra, Göttingen, Germany), gels were stained with PageBlue™ Protein Staining Solution (Thermo Fisher Scientific, 24620, Vilnius, Lithuania).

### 2.12. Western Blot

After separation by SDS-PAGE, proteins were electrophoretically transferred (1 mA/cm^2^ blot for 40 min) (Biometra, Fastblot B33 846-014-100, Göttingen, Germany) to a polyvinylidene difluoride (PVDF) membrane (Carl Roth, T830.1). The membrane with the transferred proteins was blocked with 2% milk powder (Carl Roth, T145.3) in PBS for 1 h at RT or overnight at 4 °C. Then the PVDF membrane was rinsed twice with PBS-T and incubated with hybridoma supernatants (undiluted) or with the MAb M75 to human CA IX (BioScience, Bratislava, Slovakia) diluted 1:500 for 1 h at RT. After washing in PBS-T, goat anti-mouse IgG antibody conjugated to HRP (Bio-Rad, 1721011 Hercules, CA, USA), diluted to 1:4000 in PBS-T, was added and incubated for 1 h at RT. The membrane was rinsed with PBS-T 5 times and the bands of proteins were visualized using 1-Step™ Ultra TMB-Blotting Solution (Thermo Scientific, 37574, Rockford, IL, USA) for 5 to 10 min or membranes were covered for 10–45 min in freshly prepared 4-chloro-1-naphthol solution: 2 mL of methanol stock solution (1 tablet of 4-Chloro-1-naphthol (Merck KGaA, C6788-50TAB, St. Louis, MO, USA) dissolved in 10 mL of methanol (Carl Roth, T169.1, Karlsruhe, Germany)), 10 mL PBS, and 30 μL of 30% hydrogen peroxide (Carl Roth, 9681.1, Karlsruhe, Germany). Afterwards, membranes were washed in distilled water, dried, and scanned.

### 2.13. Flow Cytometry Analysis

Cell cultures were grown under normoxic or hypoxic conditions to confluence of 90% and harvested. The cells (1 × 10^6^ cells per test) were suspended in 200 µL of undiluted hybridoma supernatants and incubated for 1 h at RT in the dark. Cells were washed with 1 mL dilution/washing buffer (1% FBS and 0.1% sodium azide (Merck KGaA, 71289-250G) in PBS), collected by centrifugation and incubated for 30 min at 4 °C in the dark with Goat anti-mouse IgG (H+L) highly cross-adsorbed secondary antibody Alexa Fluor 488 (Thermo Fisher Scientific, A-11029). After washing, the cells were fixed for 15 min at 4 °C in the dark with BD Cytofix Fixation Buffer (BD Biosciences, 554655). Cells were rinsed and transferred to dilution/washing buffer until analysis. Prepared cells were analyzed using CyFlow Space flow cytometer (Sysmex Partec, CY-S-3001, Norderstedt, Germany). More than 30,000 events per test were evaluated with FloMax 2.7 software. Flow cytometry data were analyzed with FlowJo (FlowJo, LLC, Ashland, Oregon, USA). As an isotype control, irrelevant MAbs of IgG1 subtype prepared in-house against porcine parvovirus (PPV) were used [42].

### 2.14. Immunoprecipitation

Cells were grown in 75 cm^2^ cell culture flasks (Nunc, Thermo Fisher Scientific, 156499, Roskilde, Denmark) under normoxic or hypoxic conditions. Cell culture medium was poured off and cells were washed with PBS buffer. 1 tablet of protease inhibitor (Pierce, Thermo Fisher Scientific, A32963, Rockford, IL, USA) was dissolved in lysis buffer (150 mM NaCl, 1% NP-40 (Thermo Scientific, 85124), 0.5% sodium deoxycholate (C_24_H_40_O_4_) (Carl Roth, 3484.3, Karlsruhe, Germany), 0.1% sodium dodecyl sulphate (SDS) (Carl Roth, 2326.2, Karlsruhe, Germany), 50 mM Tris(hydroxymethyl)-aminomethane (TRIS) (Carl Roth, 4855.2, Karlsruhe, Germany)), and 1 mL of prepared mixture was then added to the cell culture flask, incubated for 30 min at 4 °C. The lysate was centrifuged at 20,000× *g* for 15 min and the separated supernatant was used for immunoprecipitation (IP) or stored at −20 °C.

Five mg of MAb H7 and 500 µL rProtein A Sepharose Fast Flow (GE Healthcare Life Sciences, 17127901, Uppsala, Sweden) were washed 5 times with 0.1 M Tris-HCl solution and each time centrifuged at 3000× *g* for 5 min. The prepared MAb and rProtein A Sepharose were mixed together and incubated for 2 h at RT with gentle rotation. After incubation, the mixture was centrifuged at 3000× *g* for 5 min. Then the MAb-sepharose complex was equally divided to the cell lysates and the control sample (2 µg of recombinant CA IX^+PG^ protein) and incubated over night at 4 °C with gentle rotation. Collected samples were centrifuged at 3000× *g* for 5 min and washed 4 times with PBS, each time centrifuged at 3000× *g* for 5 min, and resuspended in 100 µL PBS. The immunoprecipitated proteins were analyzed by SDS-PAGE (30 µg of cell lysates, control and purified MAb per lane) and WB, using CA IX specific HRP labeled MAb H7 diluted 1:1000 in PBS-T buffer.

### 2.15. Immunofluorescence Staining

For the immunofluorescence assay (IFA), HeLa cells were seeded on glass coverslips in 12-well culture plates and incubated under hypoxic conditions for 48 h. Cells were fixed by submerging glass coverslips in −20 °C methanol for at least 10 min. After rehydration in PBS supplemented with 0.05% of Tween 20 (PBS-TW), cells were incubated with the undiluted supernatants of all 13 hybridoma cell lines and isotype control hybridoma (irrelevant PPV-specific MAb of IgG1 subtype) for 30 min at 37 °C. After incubation, cells were washed in PBS-TW for 30 min by changing buffer 3 times and incubated with secondary Alexa Fluor 488-labeled antibody (Thermo Fisher Scientific, A-11029, Eugene, OR, USA) diluted in PBS-TW (1:200) for 30 min at 37 °C. After washing with PBS-TW for 30 min, glass coverslips were additionally washed in distilled water and mounted on the microscope slides by placing cells down on the drops of ProLong Diamond Antifade mountant with DAPI (Thermo Fisher Scientific, P36971). Cell microscopy and photography was performed using EVOS FL auto microscope (Thermo Fisher Scientific, AMAFD1000, Bothell, WA, USA).

MDA-MB-231 cells were grown on collagen pre-coated glass coverslips and incubated under normoxic or hypoxic conditions. After 48 h, cells were fixed with 4% paraformaldehyde in PBS, methanol free (Thermo Scientific Pierce, 28908, Rockford, IL, USA). Nonspecific binding was blocked by incubation with PBS (Gibco, Thermo Fisher Scientific, 10010015, Waltham, MA, USA) containing 2% BSA, 2% FBS (GE Healthcare Life Sciences HyClone Laboratories, SH30071, Pittsburgh, PA, USA) and 0.4% saponin (Merck KGaA, 47036, Darmstadt, Germany). Cells were incubated with an undiluted hybridoma supernatant of CA IX-specific MAb H7 or the isotype control (irrelevant PPV-specific MAb of IgG1 subtype) for 1 h, then incubated with goat anti-mouse Alexa Fluor 488-conjugated IgG (H+L) antibody (Thermo Fisher Scientific, A28175, Waltham, MA, USA) and Alexa Fluor 568 Phalloidin at a concentration 1:50 (Thermo Fisher Scientific, A12380, Eugene, OR, USA) for 1 h. DAPI (Thermo Fisher Scientific, D1306, Eugene, OR, USA) was used to stain nucleus.

### 2.16. Near-Infrared Fluorescence Western Blot

MDA-MB-231 cells were grown in 10 cm tissue culture-treated plates under normoxic or hypoxic conditions. After 48 h, cells were washed with cold PBS, scraped into microcentrifuge tube and lysed on ice by adding 1% Triton X-100 solution in PBS containing 1 mM phenylmethanesulfonyl fluoride. Proteins were separated using a 10% SDS-PAGE and transferred to PVDF membranes (Millipore, Burlington, MA, USA). Membranes were incubated in 5% dry milk for 1 h then washed three times with 15 mL of Tris buffered saline with 0.5% Tween 20 (TBS-T). After blocking, membranes were incubated with the MAb H7 at 1:500 dilution and β-tubulin rabbit polyclonal antibody at 1:2500 dilution (Sigma-Aldrich, T6074, St. Louis, MO, USA) for 2 h at room temperature. After washing tree times with TBS-T, membranes were incubated with IRDye 800CW anti-rabbit (LI-COR Biosciences, 926-32213, Lincoln, NE, USA) and IRDye 680RD anti-mouse (LI-COR Biosciences, 926-68072) antibodies at 1:10,000 dilution for 20 min at room temperature. Immunoreactive proteins were detected using Odyssey Imaging System (LI-COR Biosciences). Immunoblots were quantified using Image J software (NIH, Bethesda, MD, USA).

### 2.17. Immunohistochemistry

IHC staining was performed on the formalin-fixed and paraffin-embedded tissue specimens of cervical carcinoma in situ, collected after conizations at the National Cancer institute of Lithuania. IHC staining was performed on the fully automated BenchMark ULTRA IHC/ISH Staining system (Roche) at the National Center of Pathology (Vilnius, Lithuania), using EnVision FLEX, High pH kit (Dako, Agilent, K8023, Santa Clara, CA, USA), affinity purified MAb H7 (0.02 mg/mL) and hematoxylin (Merck, 75290, Burlington, MA, USA) as a nuclear counterstain. Unrelated in-house PPV-targeting MAb of IgG1 isotype was used as negative control. Stained tissue sections were analyzed and photographed using ScanScope XT system (Aperio, Leica Biosystems Inc., Buffalo Grove, IL, USA).

## 3. Results

### 3.1. Generation of MAbs against CA IX

**Figure 1:**
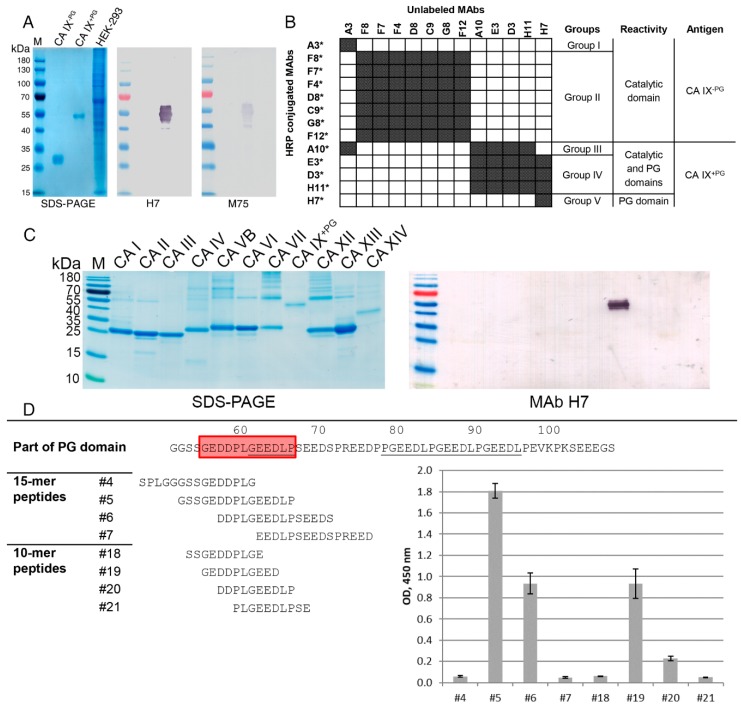
(**A**) The reactivity of MAbs H7 and MAb M75 with denaturized recombinant CA IX^-PG^ and CA IX^+PG^ proteins. Sodium dodecyl sulfate–polyacrylamide gel electrophoresis (SDS-PAGE) (the most left) and immunoblots are shown. Line M, prestained MW marker; purified recombinant CA IX^−PG^ and CA IX^+PG^ were fractionized; lysate of HEK-293 cells was used as a negative control. (**B**) Summarized results of competitive ELISA and the proposed MAb classification into groups. White box – no competition; gray box—competitive MAbs. Reactivity of MAbs with different domains and antigens used for immunizations are presented. (**C**) The reactivity of the MAb H7 with SDS-denatured recombinant CA I, II, III, IV, VB, VI, VII, IX, XII, XIII, and XIV proteins by WB (right panel). Purified recombinant proteins were fractionized by SDS-PAGE (left panel). Line M, prestained MW marker. (**D**) Representation of MAb H7 putative epitope (red box) and M75 epitope (underlined) positioning in the PG domain sequence and the overlap of synthetic peptides 5, 6, 19, and 20 recognized by MAb H7. Histogram showing the reactivity of the MAb H7 to different synthetic peptides. Optical density (OD) at 450 nm is provided.

Both the recombinant full-length extracellular domain of CA IX and recombinant catalytic domain without PG region (CA IX^+PG^ and CA IX^−PG^, respectively) were immunogenic and induced a moderate-titered antibody response in the immunized BALB/c mice (data not shown). The best responder mice from each group immunized with either CA IX^+PG^ or CA IX^−PG^ protein were selected and sacrificed for hybridoma development. After fusion, IgG-secreting clones were transferred to semisolid growth medium and screened using the robotized Clone Pix 2 system. After screening of all viable clones, a dot-plot of area against fluorescence unit (FU, exterior fluorescent intensity) of clones was generated where 1000 FU served as the threshold for selecting IgG-positive clones. Only 75 and 84 clones were above the threshold value for CA IX^+PG^ and CA IX^−PG^ antigens, respectively, and met all the criteria. Those clones were automatically picked into 96-well plate with liquid growth medium and tested by an indirect ELISA for their specificity to CA IX^+PG^ and CA IX^−PG^ proteins 3 days after transfer. In total, 13 stable hybridoma cell lines producing CA IX-specific MAbs were obtained after few rounds of recloning and additional testing (Table 2). Eight of them were raised against recombinant CA IX^−PG^ protein, indicating their specificity to the catalytic domain of CA IX and 5 MAbs were generated against recombinant CA IX^+PG^ protein (Figure 1B).

### 3.2. Characterization of MAbs by Various Immunoassays

An indirect ELISA was used in the first stage of MAb characterization to determine the specificity and affinity of MAbs to their respective antigens as well as investigate their possible cross-reactivities with other CA isoforms. The analysis of MAb reactivity with CA IX^+PG^ and CA IX^−PG^ antigens revealed three clusters of MAbs binding to three different sites of CA IX protein (Table 2, Figure 1B): (1) the catalytic domain binding MAbs, which were raised against CA IX^−PG^ and were reactive with both antigens, CA IX^+PG^ and CA IX^−PG^ (clones A3, F12, F8, F7, F4, D8, C9, and G8); (2) the catalytic and PG domains binding MAbs, which were raised against CA IX^+PG^ protein and were strongly reactive with CA IX^+PG^, but much weaker reactive with CA IX^−PG^ (clones E3, A10, H11, and D3), indicating that amino acid (aa) sequences involved in composing their epitopes are located in both catalytic and PG domains of CA IX; and (3) exclusively the PG domain targeting MAb H7 that did not recognize CA IX^−PG^ protein, which indicates epitope localization in the PG domain.

The binding strength between the generated MAbs and recombinant CA IX proteins defined as an apparent dissociation constant (K_d_) ranged from 1.31 × 10^−10^ M to 8.85 × 10^−10^ M, indicating that all antibodies are of high affinity (Table 2). In addition, the apparent K_d_ of the commercially available MAb M75 to human CA IX was determined. It was equal to 9.77 × 10^−10^, showing similar binding strength as compared with the novel MAbs.

The cross-reactivity of all MAbs with other CA isoforms (CA I, II, IV, VB, VI, VII, XII, XIII, and XIV) was investigated by indirect ELISA using recombinant proteins as antigens [36,37,38]. No cross-reactivity with other CAs was observed, which indicates high specificity of the generated MAbs to CA IX (Table S1).

The ability of the MAbs to detect sodium dodecyl sulfate (SDS)-denatured antigens was studied by Western blot (WB). As a negative control, the lysate of human embryonic kidney 293 cell line (HEK-293) cells used as producers of recombinant antigens was loaded to avoid antibodies raised against unintended proteins that possibly remained after antigen purification. As a positive control, MAb M75 was used to confirm a proper structure of the in-house produced recombinant CA IX antigens. As expected, the MAb M75 was reactive with denatured CA IX^+PG^ protein (Figure 1A) as it recognizes a linear epitope in the PG domain [43] and no reaction was observed with CA IX^−PG^, which lacks the PG domain. From all our MAb collection only the MAb H7 recognized the denatured CA IX, in particular, CA IX^+PG^ protein, which confirms the results obtained by an indirect ELISA. Thus, the linear epitope of the MAb H7 is localized in the PG domain, similarly to the MAb M75. The MAb H7 did not show any cross-reactivity with cell lysate proteins, which verifies its specificity (Figure 1A). It was concluded that all other MAbs recognize conformational epitopes as they did not react with recombinant CA IX under denaturing conditions. MAb H7 was also tested for cross-reactivity with denatured recombinant proteins of other isoforms of CA (CA I, II, III, IV, VB, VI, VII, XII, XIII, and XIV) by WB and no reaction was observed (Figure 1C), indicating unique epitope recognized by this MAb.

### 3.3. Epitope Analysis by a Competitive ELISA and an Indirect ELISA Using Overlapping Peptides

Epitope characterization was performed by a competitive ELISA to determine whether the MAbs interfere with each other for epitope binding or they bind to different parts of CA IX. Shortly, the principle of the competitive ELISA was as follows: after binding of the unlabeled MAb to the immobilized antigen, the second MAb conjugated with HRP was added to determine if the first MAb bound to the antigen prevents binding of the HRP-labeled MAb. The MAbs were conjugated with HRP and all possible combinations of the unlabeled and labeled MAbs were tested.

The MAbs were arranged into five groups according to their overlapping and nonoverlapping epitopes (Figure 1B). The MAbs generated against the catalytic domain of CA IX were separated into two groups: the MAb A3 did not show any competition with other antibodies (group I), while other MAbs (clones F8, F7, F4, D8, C9, G8, and F12) were competing with each other, which indicates the same or adjacent binding site (group II). The MAbs generated against CA IX^+PG^ protein were divided into further three groups: MAbs recognizing conformational epitopes potentially consisting of aa sequences from the catalytic and the PG domains interfered with each other and were classified to group IV (clones E3, D3, and H11); the MAb A10 was also competing with MAbs from group IV, however it was classified to a separate group III because its binding to CA IX^+PG^ protein was inhibited by the MAb A3. We speculate that the epitopes of the MAbs belonging to groups I, III, and IV might be overlapping or adjacent, but not identical. The MAb H7 was classified to a separate group V as no competition with other MAbs was observed, indicating that it interacts with a unique epitope.

More accurate epitope mapping of the MAb H7 was performed using a set of 15 overlapping synthetic biotin-conjugated 15-mer peptides covering 38–112 aa sequence of the PG domain (Table 1, peptides 1–15). An overlap of each peptide was done by 5 aa with an offset of 5 aa, thus the accuracy in predicting the epitope was 10 aa residues. The MAb H7 recognized both peptide 5 and peptide 6, however a stronger interaction with peptide 5 was determined (Figure 1D). This suggested that the MAb H7 recognizes the epitope 57-DDPLGEEDLP-66. In contrast, the previously described MAb M75 [43] recognized peptide 6, but not peptide 5 (data not shown).

To identify the sequence of MAb H7 epitope, additional 9 overlapping biotin-conjugated 10-mer peptides with an overlap of eight aa (offset of 2 aa) were generated (Table 1, peptides 16–24). These peptides comprised the PG domain sequence from aa 46 to 76 including the identified H7 epitope. The MAb H7 showed a moderate reactivity with peptide 19 and a weak reactivity with peptide 20 (Figure 1D). This suggests that the epitope of H7 is longer than 10 aa and eventually can be specified as aa sequence 55-GEDDPLGEEDLP-66 since the absence of N-terminal glycine and glutamic acid residues considerably affects the affinity of the MAb H7 to the peptide. Also, C-terminal truncated peptides (Table 1, peptides 25–28) were tested and no reactivity of these peptides with the MAb H7 was observed. This confirms that longer epitope is required for MAb H7 and CA IX PG domain interaction.

### 3.4. Application of the MAbs for Studying CA IX Expression in Human Tumor Cells

Eight human tumor cell lines representing various cancer types (Table 3) were grown either in normoxic or hypoxic conditions to evaluate the applicability of the newly generated MAbs for studying CA IX expression. Flow cytometry was applied to determine the interaction of the MAbs with native CA IX protein located on the surface of live cells. The MAbs were also tested for their ability to isolate native CA IX from cell lysate by IP.

**Figure 2:**
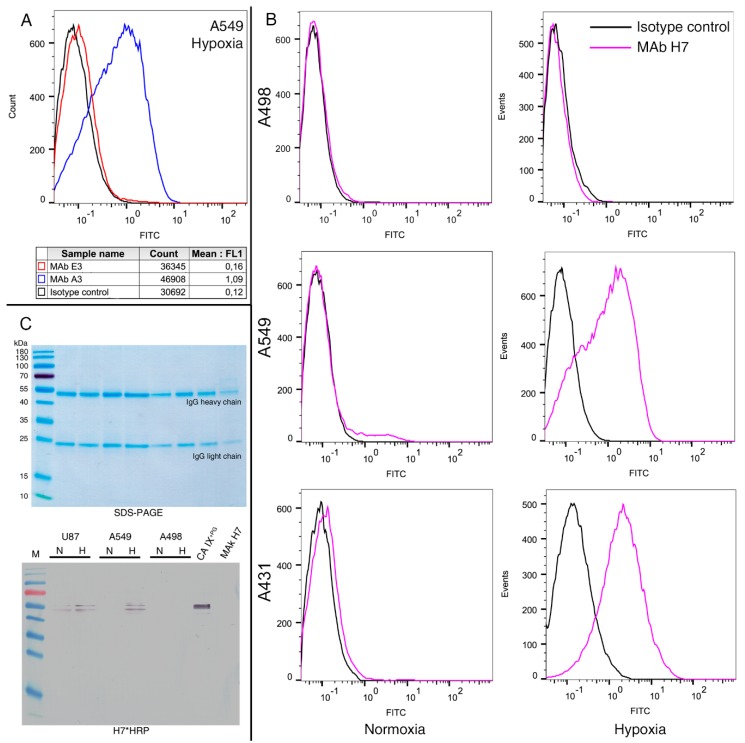
(**A**) Flow cytometry fluorescence histograms of A549 cells grown in hypoxia and stained with anti-CA IX MAbs to determine interaction of MAbs with native CA IX protein. Positive MAb A3 was compared with negative clone E3 and isotype control (irrelevant IgG1 subclass in-house MAb). (**B**) CA IX expression represented by flow cytometry fluorescence histograms of A498, A549 and A431 cells grown in normoxia and hypoxia and stained with MAb H7. Histograms demonstrate the rightward shift for the CA IX positive signal compared to isotype control. (**C**) CA IX protein immunoprecipitation from U-87, A549, and A498 cell lysates and detection by WB. Cells were cultivated in normoxic (N) and hypoxic (H) conditions. Top panel: SDS-PAGE—heavy and light chains of MAb H7 used for immunoprecipitation are seen. Lower panel: WB with HRP-labeled MAb H7 (H7*HRP). Line M, prestained MW marker; recombinant CA IX^+PG^ protein was precipitated under identical conditions and was used as positive control. MAb H7 alone was used as negative control.

Human lung carcinoma cell line (A549) with known CA IX expression under hypoxic conditions [44] was selected to investigate the reactivity of the MAbs with native extracellular domain of CA IX by flow cytometry. Increased fluorescence intensities were observed after incubating A549 cells with eight MAbs raised against CA IX^−PG^ (clones F12, G8, F7, F4, D8, C9, A3, and F8) and the CA IX^+PG^-specific MAb H7 (Figure 2A). Four MAbs, three of them A549-positive (clones A3, F4, and H7) and one clone A549-negative (E3), were further selected to analyze CA IX expression by flow cytometry in different human tumor cells (cell lines A549, U-87, A498, MCF-7, A431, CaSki, HeLa, and Jurkat) grown under normoxic and hypoxic conditions. In total, four cell lines did not show CA IX expression under regular oxygen concentration; however, after 7 days of cultivation in hypoxic environment the MAbs, clones A3, F4, and H7 were able to detect CA IX on the surface of these cells. In contrast, two cell lines (U-87 and CaSki) had detectable CA IX expression levels in normoxia while fluorescence intensity shifts were observed in hypoxia. No MAb reactivity was detected with A498 and Jurkat cells indicating that these cells do not express CA IX (Table 3, Figure 2B).

The only MAb H7 reactive with denatured recombinant CA IX was applied for the IP experiments for isolating CA IX from cell lysates of all selected tumor cells grown in normoxia and hypoxia. The prepared cell lysates were incubated with the MAb H7 and Protein A-Sepharose for CA IX IP using recombinant CA IX^+PG^ protein as a positive control. After SDS-PAGE and WB analysis of the immunoprecipitates using HRP-conjugated MAb H7, the precipitated CA IX protein was identified as a protein band of 54 kDa (Figure 2C). Analysis of all cell lines revealed highly consistent results of immunoprecipitation and flow cytometry (Table 3).

### 3.5. Application of the MAbs for the Visualization of CA IX-Positive Tumor Cells by Immunofluorescence

**Figure 3:**
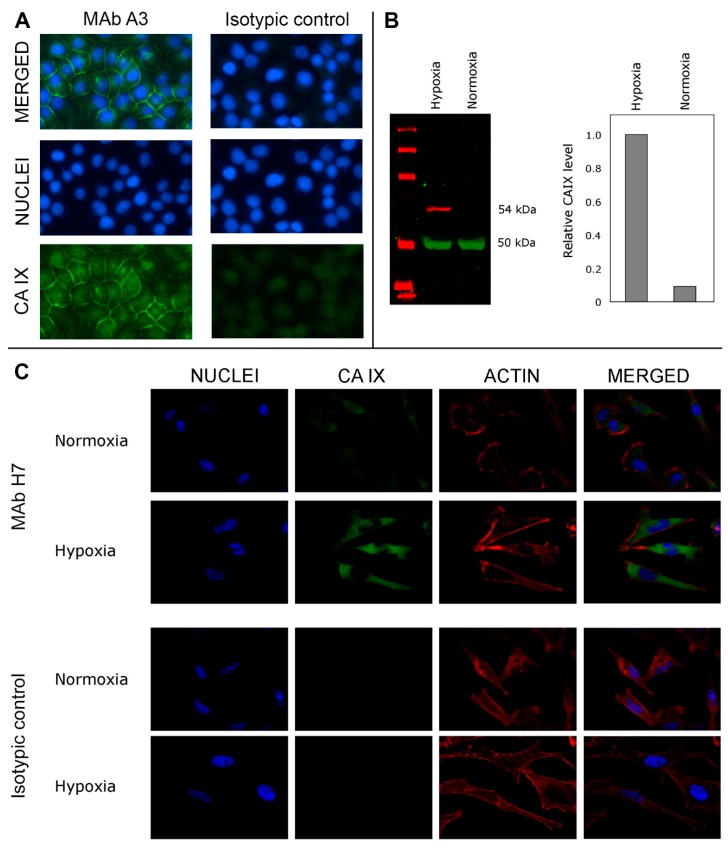
(**A**) Visualization of CA IX expressed in HeLa cells grown under hypoxia by immunofluorescence using anti-CA IX A3 and secondary anti-mouse IgG Alexa Fluor 488 antibody. Fluorescence microscopy images were taken at a ×20 magnification. (**B**) Characterization of anti-CA IX H7 using near-infrared fluorescence Western blot in triple-negative breast cancer cell line MDA-MB-231 cells under normoxic and hypoxic conditions. Human carbonic anhydrase IX was identified using MAb H7 at a dilution of 1:500. (**C**) Characterization of antibody H7 by immunofluorescence in MDA-MB-231 cells under normoxic and hypoxic conditions. CA IX was identified using MAb H7 and Alexa Fluor 488 conjugated goat anti-mouse IgG. Actin was stained using Alexa Fluor 568 Phalloidin, and nuclei were stained using DAPI. Fluorescence microscopy images of cells were taken at a ×63 magnification.

All MAbs were tested in immunofluorescence microscopy for imaging HeLa cells grown in hypoxia for 48 h. After methanol fixation and rehydration in PBS, cells were incubated with primary MAbs following incubation with secondary anti-mouse IgG Alexa Fluor 488 antibody. Several MAbs showed a moderate reactivity (clones G8, E3, H11, and D3), while others were strongly positive (clones A3, F12, F8, F7, F4, D8, C9, H7, and A10, Table 1) demonstrating expression of membrane-bound and cytoplasmic CA IX (Figure 3A).

The MAb H7 was also tested for the immunostaining of breast cancer MDA-MB-231 cells and revealed much higher CA IX expression levels under hypoxic conditions compared to normoxia (Figure 3C). In addition, highly sensitive near-infrared fluorescence Western blot was applied to analyze MDA-MB-231 cells grown in normoxia and hypoxia allowing a direct CA IX detection in cell lysate with the MAb H7 without IP. This method reliably confirms the specificity of the MAb H7 and allows quantitative calculations of CA IX expression (Figure 3B).

### 3.6. Development of Sandwich ELISA for Detection of Soluble CA IX

**Figure 4:**
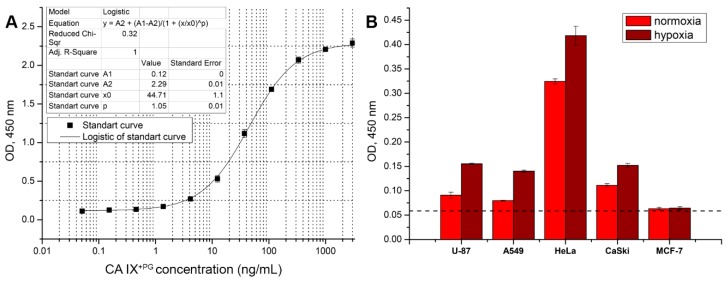
(**A**) Calibration curve obtained after optimization with selected A3 and HRP-labeled MAb H7 pair. Recombinant CA IX^+PG^ protein was used as a standard. (**B**) Application of developed sandwich ELISA for the detection of the soluble CA IX in the culture media of various cancer cells after 10 days cultivation in the normoxic and hypoxic conditions. Dashed line represents background level.

To analyze the soluble CA IX form in cell growth medium or human body fluids, a two-epitope (sandwich) ELISA was developed. The appropriate pair of the MAbs targeting different parts of CA IX was selected based on the results of a competitive ELISA. The capture antibody was immobilized on the 96-well plate and the detection antibody was conjugated with HRP for signal development. Recombinant CA IX^+PG^ was used for selecting the best MAb pair, optimization of assay conditions, and as a standard for CA IX quantification. Taking into account the results obtained previously by competitive ELISA, in total 33 combinations of different MAbs were tested and two pairs (capture MAb A3 and HRP-H7, and capture MAb F12 and HRP-H7) showing the highest binding sensitivity (data not shown) were selected for the development of two-epitope ELISA. In all tested combinations, the MAb H7 demonstrated the best performance as a detection antibody. After testing different ELISA conditions (plate types, blocking solutions, incubation temperature and time, concentration of capture, and detection antibodies) the capture MAb A3 and HRP-labeled MAb H7 showed the best sensitivity for CA IX^+PG^ detection reaching 0.111 ± 0.046 ng/mL (Figure 4A).

The newly developed sandwich ELISA was applied for the quantification of soluble CA IX shedded into growth medium of tumor cells grown for 10 days in normoxia and hypoxia. The measured concentrations of CA IX in the growth medium ranged from 0.97 ng/mL, for U-87 cells cultivated in normoxia, to 31.67 ng/mL, for HeLa cells grown in hypoxia. Soluble CA IX was not detected in the supernatants of Jurkat, A498 and MCF-7 cells (Figure 4B).

Sandwich ELISA was also applied for CA IX quantification in human plasma specimens obtained from 14 patients with cervical pathology. Six specimens (42.86%) out of 14 were found to be CA IX-positive with CA IX concentrations ranging from 0.31 to 10.1 ng/mL (data not shown). These results demonstrate the reactivity of the MAbs with native soluble CA IX present in human blood and the potential of the newly developed ELISA to detect CA IX in biological samples.

### 3.7. Application of the MAbs for CA IX Detection in Tissues by the Immunohistochemistry Assay

**Figure 5:**
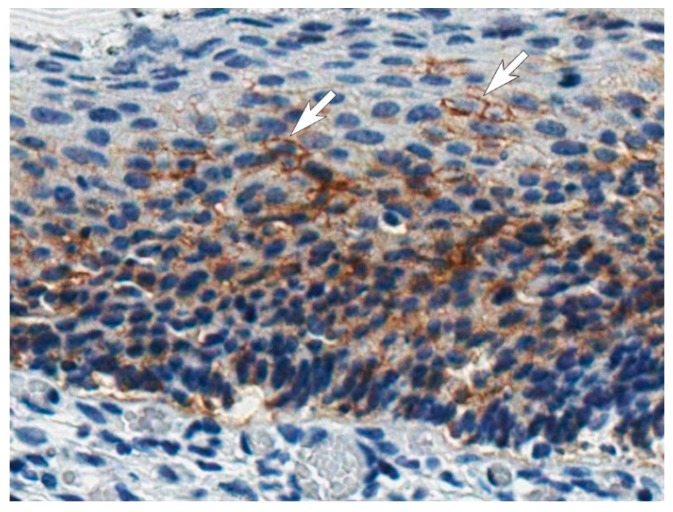
Immunohistochemical CA IX detection in the tissue section of cervical carcinoma in situ using MAb H7. Staining of the cellular membranes is evident in several regions (shown by white arrows). Magnification × 40.

All MAbs were examined for the detection of CA IX expression in cervical carcinoma in situ specimens by IHC technique. Formalin-fixed and paraffin-embedded tissue sections were immunostained according to the protocol previously developed for the detection of CA IX with anti-CA IX antibodies (clone EP161, Cell Marque, USA) at the National Center of Pathology (Vilnius, Lithuania) to confirm CA IX expression and then analyzed with the newly generated MAbs (data not shown). A clear positive staining of CA IX was observed only with the MAb H7, which corelates with WB data indicating that only this MAb is capable to recognize the denatured CA IX. A representative IHC picture of CA IX-positive cervical carcinoma is shown in Figure 5. This lesion is characterized by cellular abnormality, which prevails throughout the full thickness of the epithelium: an increased nuclear–cytoplasmic ratio, nuclear enlargement and hyperchromatic staining, and mitoses present throughout the lower/middle third of the epithelium. CA IX was detected in the membrane of carcinoma cells (highlighted with white arrows in Figure 5). No signs of any nonspecific staining were observed confirming high specificity of the MAb H7 and its potential for CA IX detection by IHC.

## 4. Discussion

Antibodies are important and attractive tools in medicine and biology because of their specificity, flexibility and versatility to be adapted in a variety of methods [45]. Thousands of MAbs targeting various proteins are currently in the market for research, diagnostics, or therapy [46,47]. However, development of highly specific and well-characterized MAbs is a challenging and time-consuming process. It is now admitted, that many commercially available MAbs lack specificity or some results are difficult to reproduce, thus indicating a demand for new broadly characterized antibodies [48].

Disease-related antigens are major targets for the MAbs produced in recent years, in particular those developed for cancer diagnostics and therapy [7,12]. Several MAbs generated against cancer-related enzyme CA IX are widely used in many studies proving CA IX as a reliable diagnostic and prognostic biomarker of cancer as well as a potential target for cancer therapy. For many years, two MAbs G250 and M75 against CA IX were the most applicable in the fields of therapy and diagnostics [49]. Despite this fact, the properties of MAb G250 were investigated in more detail only recently [50]. In the current study, we present a new collection of well-characterized MAbs targeting CA IX, which could supplement and potentially improve research in the field of CA IX-related cancers.

A few unsuccessful attempts to generate high-affinity MAbs targeting CA IX were made by our group using standard procedures of MAb production including immunization, cell fusion, and clone selection (data not shown). The main problem was related to an insufficient immune response in mice and low level of specific antibodies against recombinant CA IX used as an immunogen, possibly due to its high homology (~70%) to murine CA IX. To improve the efficiency of immunization and generate MAbs targeting different epitopes of human CA IX, we immunized mice with recombinant proteins representing the catalytic domain of CA IX with or without the PG domain (CA IX^+PG^ and CA IX^−PG^, respectively). The recombinant CA IX antigens were produced in human HEK-293 cells expecting high similarity of both antigens to the native CA IX in terms of post-translational modifications. Screening procedure of IgG-producing hybrid clones was performed with a robotic clone imaging and picking module to increase screening efficiency. Further steps involved screening of clones by an indirect ELISA to select CA IX-specific clones and generate stable hybridoma cell lines. Thirteen hybridoma clones secreting CA IX specific-MAbs were selected and the MAbs were subjected to a subsequent characterization by different immunoassays.

All selected MAbs were of IgG1 subclass, showed high affinity to CA IX and did not react with other CAs. Most of the MAbs (eight out of 13) were reactive with native CA IX on cell surface and recognized conformational epitopes. The MAb H7 reactive with cell surface CA IX and specific for the PG domain distinguished from other MAbs by recognizing a linear epitope, which was mapped at aa 55–66 of the PG domain (55-GEDDPLGEEDLP-66). In contrast, other MAbs were raised against the catalytic domain or probably shared aa residues from both PG and catalytic domains. Our study demonstrated wide application possibilities of the MAb H7 which was reactive with both native and denatured CA IX by flow cytometry, IP, immunofluorescence, near-infrared fluorescence Western blot, IHC, and sandwich ELISA.

The MAb H7 was selected as a detection antibody in a pair with the capture MAb A3 to develop sandwich ELISA for the detection of soluble CA IX shedded of the tumor cells by metalloproteases [32]. The ELISA was applied to detect CA IX in the supernatants of several tumor cell lines cultured under normoxia and hypoxia and blood plasma specimens of 14 individuals. Although an additional ELISA improvement is needed to increase its sensitivity, this assay allowed detection of CA IX in growth medium of tumor cell lines and some blood plasma specimens. The interest in the shedding of CA IX ectodomain is increasing and various cancer cell lines and specimens of cancer patients are under investigation. The first observation of the soluble CA IX form was in growth medium of HT29 (colorectal carcinoma) cells grown in normoxia. In contrast, CA IX was not detected in A498 cell line [31]. Later it was found that CA IX ectodomain shedding depends on metalloproteases and increases in response to hypoxia. This augmentation was also associated with an increased amount of membrane CA IX and was observed in CaSki, SiHa (cervical carcinoma), ACHN (renal cell carcinoma), HeLa, HT-29, CGL1, and CGL3 (hybrids between HeLa and normal fibroblasts) cells [32]. Our results confirmed these findings in A498, HeLa and CaSki cells. In addition, novel results indicating an increase in CA IX shedding in A549 and U-87 cells under hypoxic conditions were obtained.

Recent studies have brought more understanding in terms of biological meaning of ectodomain shedding. If treated with cytotoxic drugs, apoptotic cells increase shedding of the extracellular CA IX domain. Emerging evidence reveals a paracrine signaling function of circulating CA IX. It is speculated, that shedded CA IX ectodomain affects the adjacent cancer cells and they can acquire a chemotherapy-resistant phenotype [51]. In addition, there is still a lack of sufficient evidence of the clinical value of soluble CA IX, so the emergence of new diagnostic tools would improve the field. The developed ELISA test based on the selected pair of anti-CA IX MAbs H7 and A3 was suitable to detect the ectodomain of CA IX in blood plasma specimens thus providing a potential tool for further CA IX studies.

Application possibilities of the developed MAbs, in particular clone H7, were also proven in other antibody-based techniques. The MAb H7 was suitable to analyze cell surface-located CA IX by flow cytometry and to immunoprecipitate CA IX protein from cell lysates, which allowed to observe changes in protein expression level due to hypoxia effect. Highly consistent results were obtained with both techniques thus demonstrating the reliability of the used MAb. Cell lines representing lung, brain, renal, T cell, cervical, skin, and breast cancers were different in CA IX expression levels under normoxia and after the exposure to hypoxia. It was shown that Jurkat and A498 cells do not express CA IX regardless of oxygen conditions. This is in line with previous studies that did not find CA IX expression in malignant hematopoietic cells, including Jurkat cell line [52], while A498 cell line was characterized as a poor producer of CA IX [31]. The majority of investigated cell lines (A549, MCF-7, A431, and HeLa) did not express CA IX while cultured in normal oxygen concentration. However, after growing in hypoxia, these cells started expressing CA IX and this correlates well with previous studies where cell lines or specimens of the same type cancer were analyzed [44,53,54,55]. Two cell lines U-87 and CaSki were characterized by a constitutive CA IX expression in a normoxic environment and an increased CA IX level in hypoxia [32,56]. These results were confirmed with the MAb H7 by immunoprecipitation of U-87 and CaSki lysates.

Visualization of cancer cells is important for understanding of many cancer-related processes, therefore antibody-based visualization techniques are important in the field of cancer research and diagnostics [7]. We have shown the applicability of the MAbs in the IFA of HeLa cells grown in hypoxia where both membrane and cytoplasmic staining was seen. Under cultivation in hypoxia, an increase in CA IX expression was observed by the IFA and confirmed by an indirect near-infrared fluorescence Western blot in breast cancer MDA-MB231 cells using the MAb H7. The IHC analysis of CA IX expression in cervical carcinoma in situ specimen demonstrated the relevance of the MAb H7 for the detection of CA IX-positive tissues. No other clone from the newly generated MAb collection was reactive with formalin-fixed and paraffin-embedded tissue sections, thus MAb H7 has unique features and exceptionally broad application possibilities.

As the MAb H7 was shown to interact with the PG domain of CA IX, we have identified the aa sequence involved in the antibody–antigen interaction. Fine epitope mapping is important for MAb specificity validation and identification of differences between the MAbs used in CA IX assays. The MAb H7 showed similar properties with a widely used MAb M75, thus more accurate epitope analysis was applied to identify their differences [7,57]. A set of overlapping synthetic biotinylated peptides dividing the PG domain into smaller segments was selected to analyze the localization of linear epitopes of the MAbs H7 and M75. As expected, MAb M75 was reactive with synthetic peptides containing CA IX sequence (P)GEEDLP, which is repeating several times in the PG domain and was previously identified as MAb M75 epitope [43]. MAb H7 recognized peptides 5 and 6, thus an overlapping sequence 57-DDPLGEEDLP-66 was identified as a potential epitope. Both MAb H7 and MAb M75 recognized peptide 6 (DDPLGEEDLPSEEDS). However, the MAb H7 did not react with other peptides containing the GEEDLP sequence, suggesting that these aa residues are not essential for antibody–antigen interaction. The exact MAb H7 epitope was determined by testing with additional overlapping peptides and was identified as 12 aa-long sequence 55-GEDDPLGEEDLP-66. The identified aa sequence was aligned with aa sequences of other CA isoforms and no similarity was observed (data not shown) as the PG domain is unique for CA IX [18]. This confirms high selectivity of the MAb H7 for CA IX. The employment of the MAb H7 in xenografted mice for the analysis of CA IX expression may be also considered as the similarity of H7 epitope with mouse proteins is mild and the inter-species cross-reactivity is not expected [58].

Taken together, a new collection of CA IX-specific MAbs was raised against the catalytic domain of CA IX with or without the PG domain. The MAbs were characterized by different immunoassays and their selectivity for CA IX was demonstrated. The MAb of clone H7 that recognizes a linear epitope within the PG domain was extensively investigated for its applicability to detect CA IX by various antibody-based techniques including flow cytometry, IP, IHC, immunofluorescence microscopy, sandwich ELISA, Western blot. No restrictions in terms of its use to detect the native, denatured, soluble, or cell-bound CA IX were identified, which confirms its potential for diagnostics, research and possibly future therapeutic applications.

## Figures and Tables

**Table 1:** Sequences of synthetic peptides used for epitope mapping.

Peptide No.	Peptide Sequence	Peptide No.	Peptide Sequence
#1	Biotin-SGSGLVPVHPQRLPRMQED-OH	#16	Biotin-SGSGLGGGSSGEDD-OH
#2	Biotin-SGSGPQRLPRMQEDSPLGG-OH	#17	Biotin-SGSGGGSSGEDDPL-OH
#3	Biotin-SGSGRMQEDSPLGGGSSGE-OH	#18	Biotin-SGSGSSGEDDPLGE-OH
#4	Biotin-SGSGSPLGGGSSGEDDPLG-OH	#19	Biotin-SGSGGEDDPLGEED-OH
#5	Biotin-SGSGGSSGEDDPLGEED-OH	#20	Biotin-SGSGDDPLGEEDLP-OH
#6	Biotin-SGSGDDPLGEEDLPSEEDS-OH	#21	Biotin-SGSGPLGEEDLPSE-OH
#7	Biotin-SGSGSEEDLPSEEDSPREED-OH	#22	Biotin-SGSGGEEDLPSEED-OH
#8	Biotin-SGSGSEEDSPREEDPPGEE-OH	#23	Biotin-SGSGEDLPSEEDSP-OH
#9	Biotin-SGSGPREEDPPGEEDLPGE-OH	#24	Biotin-SGSGLPSEEDSPRE-OH
#10	Biotin-SGSGPPGEEDLPGEEDLPG-OH	#25	Biotin-SGSGDDPLGEED-OH
#11	Biotin-SGSGDLPGEEDLPGEED-OH	#26	Biotin-SGSGDDPLGEE-OH
#12	Biotin-SGSGEDLPGEEDLPEVKPK-OH	#27	Biotin-SGSGDDPLGE-OH
#13	Biotin-SGSGSEEDLPEVKPKSEEEG-OH	#28	Biotin-SGSGGEDDPLG-OH
#14	Biotin-SGSGEVKPKSEEEGSLKLED-OH		
#15	Biotin-SGSGSEEEGSLKLEDLPTV-OH.		

**Table 2:** Summarized characteristics of generated MAbs against recombinant CA IX.

MAb Clone	IgG Subtype	Apparent K_d_ Value, *M*	MAbs Reactivity with CA IX ^1^					
			ELISA CA IX^−PG^	ELISA CA IX^+PG^	WB CA IX^−PG^	WB CA IX^+PG^	FC ^2^	IFA ^3^
**A3**	IgG1	1.31 × 10^−10^	+	+	−	−	+	+
**F12**		5.12 × 10^−10^	+	+	−	−	+	+
**F8**		3.29 × 10^−10^	+	+	−	−	+	+
**F7**		1.42 × 10^−10^	+	+	−	−	+	+
**F4**		6.5 × 10^−10^	+	+	−	−	+	+
**D8**		6.17 × 10^−10^	+	+	−	−	+	+
**C9**		2.51 × 10^−10^	+	+	−	−	+	+
**G8**		2.14 × 10^−10^	+	+	−	−	+	±
**H7**		8.85 × 10^−10^	−	+	−	+	+	+
**E3**		1.75 × 10^−10^	±	+	−	−	−	±
**A10**		1.75 × 10^−10^	±	+	−	−	−	+
**H11**		1.71 × 10^−10^	±	+	−	−	−	±
**D3**		2.2 × 10^−10^	±	+	−	−	−	±
**M75**		9.77 × 10^−10^	−	+	−	+	ND ^4^	ND

^1^ + strong reaction; ± moderate/weak reaction; − no reaction. ^2^ FC—flow cytometry. ^3^ IFA—Immunofluorescence assay. ^4^ ND—not done.

**Table 3:** Comparison of CA IX expression in human cancer cells grown in normoxic and hypoxic conditions determined with specific MAbs by flow cytometry and IP/WB.

Cell Line	Origin	Flow Cytometry		IP/WB	
		Normoxia	Hypoxia	Normoxia	Hypoxia
**A549**	Lung carcinoma	−	+	−	+
**U-87**	Glioblastoma	+	+	+	+
**A498**	Renal cell carcinoma	−	−	−	−
**Jurkat**	T cell leukemia	−	−	−	−
**HeLa**	Cervical adenocarcinoma	−	+	−	+
**A431**	Epidermoid carcinoma	−	+	−	+
**CaSki**	Cervical squamous cell carcinoma	+	+	+	+
**MCF-7**	Breast adenocarcinoma	−	+	−	+

+ detected; − not detected.

## Supplementary Materials

The following are available online at https://www.mdpi.com/2218-273X/9/8/304/s1. Table S1: Supplementary table S1—The cross-reactivity of the generated MAbs with other CA isoforms (CA I, II, IV, VB, VI, VII, XII, XIII, and XIV) investigated by indirect ELISA using recombinant proteins as antigens. Green color—positive reaction (optical density at 450 nm is provided). PAbs—polyclonal antibodies obtained from mouse that has been immunized with CA IX^+PG^ and used for hybridization. GM—fresh growth medium for hybridoma cultivation was used as a negative control.

## Abbreviations

A431	human epidermoid carcinoma cell line
A498	human renal cell carcinoma cell line
A549	human lung carcinoma cell line
aa	amino acid
BSA	bovine serum albumin
CA	carbonic anhydrase
CA IX	carbonic anhydrase IX
CA IX^+PG^	recombinant full-length extracellular domain of CA IX
CA IX^-PG^	recombinant catalytic domain without PG region
CA XII	carbonic anhydrase XII
CaSki	human cervical squamous cell carcinoma cell line
DMEM	Dulbecco’s modified Eagle’s growth medium
ELISA	enzyme-linked immunosorbent assay
FBS	fetal bovine serum
HAT	hypoxanthine/aminopterin/thymidine
HEK-293	human embryonic kidney 293 cell line
HeLa	human cervical adenocarcinoma cell line
HRP	horseradish peroxidase
HT29	human colorectal carcinoma cell line
IFA	immunofluorescence assay
IgG	immunoglobulin G
IHC	immunohistochemistry
IP	immunoprecipitation
Jurkat	human T cell leukemia cell line
K_d_	apparent dissociation constant
MAbs	monoclonal antibodies
MCF-7	human breast adenocarcinoma cell line
MDA-MB-231	human triple-negative breast cancer cell line
OD	optical density
PBS	phosphate-buffered saline
PBS-T	0.1% Tween 20 in PBS buffer
PBS-TW	PBS supplemented with 0.05% of Tween 20
PG	proteoglycan region of CA IX
PPV	porcine parvovirus
PVDF	polyvinylidene difluoride
RT	room temperature
SDS	sodium dodecyl sulfate
SDS-PAGE	sodium dodecyl sulfate-polyacrylamide gel electrophoresis
TBS-T	Tris buffered saline with 0.5% Tween 20
TMB	3,3′,5,5′-Tetramethylbenzidine
U-87	human glioblastoma cell line
WB	Western blot
