# Supplementary material for: New Monoclonal Antibodies for a Selective Detection of Membrane-Associated and Soluble Forms of Carbonic Anhydrase IX in Human Cell Lines and Biological Samples
Source: Biomolecules. 2019 Jul 25;9(8):304. doi: 10.3390/biom9080304 (PMC6723738; doi:10.3390/biom9080304)
Supplement: Supplementary file 1 [file biomolecules-09-00304-s001.pdf]

Supplementary table S1. The cross-reactivity of the generated MAbs with other CA isoforms (CA I, II, IV, VB, VI, VII, XII, XIII, and XIV) investigated by indirect ELISA using recombinant proteins as antigens. Green color – positive reaction (optical density at 450 nm is provided). PAbs – polyclonal antibodies obtained from mouse that has been immunized with CA IX^{+PG} and used for hybridization. GM – fresh growth medium for hybridoma cultivation was used as a negative control.

MAb clone	Recombinant CA isoforms									
	**CA I**	**CA II**	**CA IV**	**CA VB**	**CA VI**	**CA VII**	**CA IX**	**CA XII**	**CA XIII**	**CA XIV**
**A3**	0.115	0.054	0.044	0.049	0.068	0.056	2.448	0.062	0.051	0.047
**F12**	0.067	0.070	0.045	0.051	0.071	0.078	2.975	0.045	0.083	0.055
**F8**	0.079	0.063	0.047	0.060	0.053	0.071	2.865	0.075	0.071	0.059
**F7**	0.057	0.077	0.045	0.067	0.058	0.085	2.803	0.082	0.091	0.053
**F4**	0.056	0.072	0.046	0.070	0.078	0.065	2.779	0.069	0.060	0.051
**D8**	0.054	0.066	0.044	0.050	0.093	0.066	1.908	0.094	0.055	0.057
**C9**	0.076	0.078	0.043	0.049	0.091	0.071	2.813	0.054	0.057	0.049
**G8**	0.072	0.066	0.049	0.058	0.052	0.082	3.263	0.049	0.093	0.053
**H7**	0.057	0.072	0.046	0.056	0.087	0.063	2.929	0.051	0.069	0.054
**E3**	0.053	0.060	0.045	0.051	0.073	0.053	2.039	0.058	0.058	0.063
**A10**	0.055	0.059	0.046	0.054	0.072	0.058	2.550	0.071	0.068	0.058
**H11**	0.048	0.054	0.045	0.060	0.074	0.053	1.925	0.059	0.049	0.054
**D3**	0.050	0.059	0.052	0.051	0.068	0.051	2.026	0.055	0.063	0.049
**PAbs**	0.277	0.558	0.432	0.153	0.485	0.348	2.481	0.452	0.511	0.131
**GM**	0.049	0.058	0.048	0.050	0.060	0.049	0.099	0.052	0.048	0.064
